# Comparative evaluation for the globin gene depletion methods for mRNA sequencing using the whole blood-derived total RNAs

**DOI:** 10.1186/s12864-020-07304-4

**Published:** 2020-12-11

**Authors:** Jin Sung Jang, Brianna Berg, Eileen Holicky, Bruce Eckloff, Mark Mutawe, Minerva M. Carrasquillo, Nilüfer Ertekin-Taner, Julie M. Cuninngham

**Affiliations:** 1 Medical Genome Facility, Center for Individualized Medicine, Mayo Clinic, Rochester, MN USA; 2 Department of Laboratory Medicine and Pathology, Mayo Clinic, Rochester, MN USA; 3 Department of Neuroscience, Mayo Clinic, Jacksonville, FL USA; 4 Department of Neurology, Mayo Clinic, Jacksonville, FL USA

**Keywords:** mRNA-Seq, Globin gene depletion, rRNA, Whole blood

## Abstract

**Background:**

There are challenges in generating mRNA-Seq data from whole-blood derived RNA as globin gene and rRNA are frequent contaminants. Given the abundance of erythrocytes in whole blood, globin genes comprise some 80% or more of the total RNA. Therefore, depletion of globin gene RNA and rRNA are critical steps required to have adequate coverage of reads mapping to the reference transcripts and thus reduce the total cost of sequencing. In this study, we directly compared the performance of probe hybridization (GLOBINClear Kit and Globin-Zero Gold rRNA Removal Kit) and RNAse-H enzymatic depletion (NEBNext® Globin & rRNA Depletion Kit and Ribo-Zero Plus rRNA Depletion Kit) methods from 1 μg of whole blood-derived RNA on mRNA-Seq profiling. All RNA samples were treated with DNaseI for additional cleanup before the depletion step and were processed for poly-A selection for library generation.

**Results:**

Probe hybridization revealed a better overall performance than the RNAse-H enzymatic depletion method, detecting a higher number of genes and transcripts without 3′ region bias. After depletion, samples treated with probe hybridization showed globin genes at 0.5% (±0.6%) of the total mapped reads; the RNAse-H enzymatic depletion had 3.2% (±3.8%). Probe hybridization showed more junction reads and transcripts compared with RNAse-H enzymatic depletion and also had a higher correlation (R > 0.9) than RNAse-H enzymatic depletion (R > 0.85).

**Conclusion:**

In this study, our results showed that 1 μg of high-quality RNA from whole blood could be routinely used for transcriptional profiling analysis studies with globin gene and rRNA depletion pre-processing. We also demonstrated that the probe hybridization depletion method is better suited to mRNA sequencing analysis with minimal effect on RNA quality during depletion procedures.

## Background

Transcriptome profiling of peripheral whole blood samples is highly desirable for biological research, drug discovery, diagnostic testing, and developing biomarkers in clinical settings [1–4]. While microarray technologies have widely been used for such investigations [4], RNA sequencing (RNA-Seq) technology provides higher sensitivity and more complete transcriptome data. RNA-Seq data enables the investigation of novel gene expression levels, alternative splicing events, and fusion genes, all of which may be associated with disease progress, status, treatment, and underlying molecular mechanisms of disease [5–7].

Total RNA from whole blood contains a large portion of globin genes, which originate from red blood cells and accounts for 80–90% of total transcripts [4]. Previous reports revealed that the presence of globin genes may affect the quality and accuracy of gene expression profiling in microarray [8], SAGE [9], and RNA-Seq [10] analyses, particularly for those genes with lower expression levels. Thus, globin gene depletion is an essential step to obtain accurate data for transcriptome analysis. For transcriptome profiling performs in whole blood, most kits for total RNA-Seq include both rRNA and globin gene depletion steps before generating the first-strand cDNA. However, we observed significant globin and rRNA gene reads in some whole transcriptome analyses of whole blood derived total RNA, suggesting that the depletion methods may be improved. mRNA library preparation kits do not include rRNA or globin depletion as selection of poly-A+ RNA enriches for protein-coding genes and overall it is a more cost-effective and sensitive approach for gene quantification and their biological function and roles when this is the primary research goal [11]. For this reason, we used stranded mRNA-Seq to evaluate globin gene removal to assess the quality of globin-depleted RNA to quantify gene expression. The evaluation will inform RNA preparation for mRNA sequencing applications.

In this study, we evaluated two methods for globin gene removal, probe hybridization and RNase H-based enzymatic digestion. The data generated from four commercially available kits were analyzed for performance on mRNA-Seq for whole blood-derived RNA transcriptome. Our results provide information on which of the globin gene removal kit is most suitable for mRNA-Seq data analysis from whole blood samples.

## Results

**Fig. 1:**
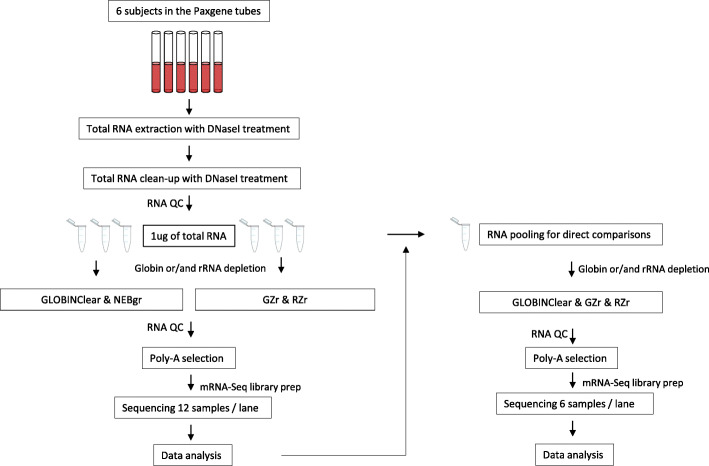
The overall experimental design is shown. Total RNA was extracted from six samples collected in Paxgene Blood Tubes and treated with DNaseI. Technical replicates of 1μg of each sample underwent depletion with one of the four kits and sequenced using the poly-A+ selection protocols. NEBgr, NEBNext® Globin & rRNA Depletion Kit; RZr, Ribo-Zero Plus rRNA Depletion Kit; GZr, Globin-Zero Gold rRNA Removal Kit

**Fig. 2:**
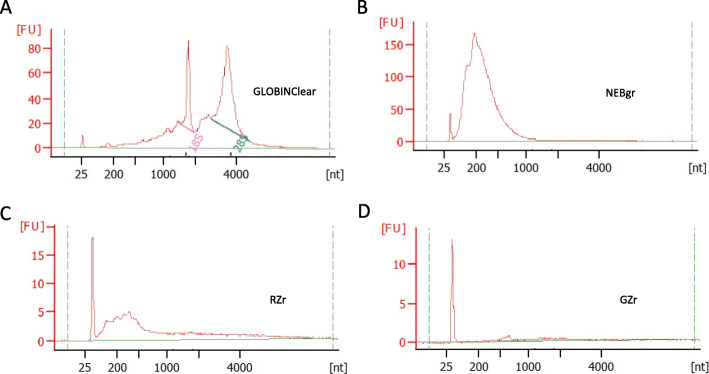
Depleted RNA QC. Total RNA depleted by the four different kits were analyzed using a Bioanalyzer 2100 High sensitivity DNA chip. **a** GLOBINClear Kit, **b** NEBgr (NEBNext® Globin & rRNA Depletion Kit), **c** RZr (Ribo-Zero Plus rRNA Depletion Kit), **d** GZr (Globin-Zero Gold rRNA Removal Kit)

Figure 1 shows the overall workflow of this study. Globin-depleted total RNA samples were checked for quality on a BioAnalyser 2100 high sensitivity DNA chip for all kits. The GLOBINClear Kit (GLOBINClear) yielded both 18 s and 28 s rRNA peak with RIN > 7.5 (Fig. 2a), while the other three kits had no rRNA peaks (Fig. 2b-d). As the GLOBINClear depleted only globin genes through probe hybridization, RNA amounts recovered were between 150 ng–200 ng, whereas the other three kits that remove both globin genes and rRNA yields were too low (less than 2 ng/ul) to be measured by Qubit. Based on the RNA peaks from the electropherogram profile in the two enzymatic depletion kits, the NEBNext® Globin & rRNA Depletion Kit (NEBgr) recovered more RNA than Ribo-Zero Plus rRNA Depletion Kit (RZr) (Fig. 2b); however, the RZr had a larger size of RNA than NEBgr (Fig. 2c). For Globin-Zero Gold rRNA Removal Kit (GZr), a probe hybridization method, RNA content could not be determined by the electropherogram profile (Fig. 2d).

**Fig. 3:**
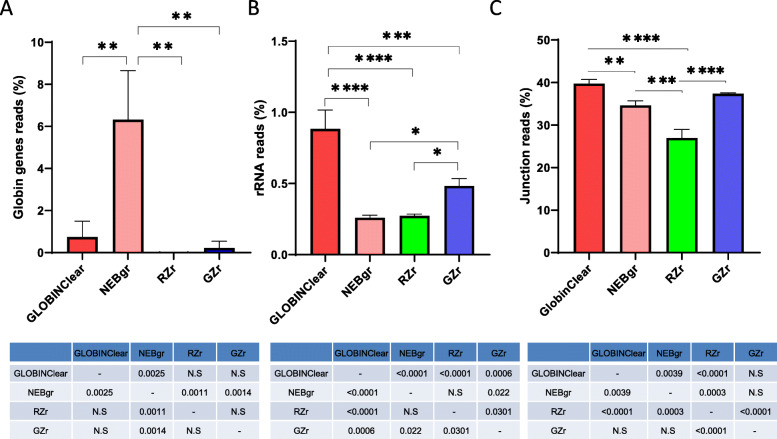
Comparison of globin gene, rRNA depletion, and junction reads across protocols. **a** Percentage of globin gene contamination in the total mapped reads, **b** Percentage of rRNA contamination in the total mapped reads, **c** Percentage of junction reads in the total mapped reads. Data are means of triplicate samples from each kit ± SD. *; *p* < 0.05, **; *p* < 0.01, ***;*p* < 0.001, ****;*p* < 0.0001. NEBgr, NEBNext® Globin & rRNA Depletion Kit; RZr, Ribo-Zero Plus rRNA Depletion Kit; GZr, Globin-Zero Gold rRNA Removal Kit; N. S, not significant

**Fig. 4:**
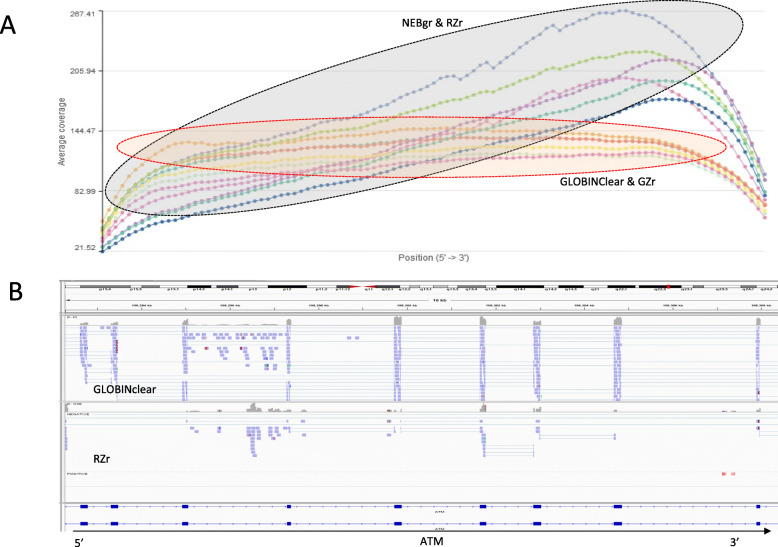
RNase-H based depletion method affected RNA quality. **a** Coverage summary plots among the four protocols. The probe hybridization method covered the entire gene body uniformly. However, the enzymatic removal method revealed skewing to 3′ region of genes. The gene body coverage plot shows samples shown as dotted lines, normalized genomic position on the horizontal axis (5′ to 3′ region of genes) and average coverage on the vertical axis. **b** Representative screenshot in the long transcript between two different depletion methods. GLOBINClear Kit covered more reads in the middle of the gene than Ribo-Zero Plus Kit. From exon 11 to 19 of the ATM gene were visualized on the IGV. NEBgr, NEBNext® Globin & rRNA Depletion Kit; RZr, Ribo-Zero Plus rRNA Depletion Kit; GZr, Globin-Zero Gold rRNA Removal Kit

The libraries generated from the four kits were sequenced to evaluate performance, particularly the efficiency of the globin gene depletion, using stranded mRNA-Seq with poly-A+ selection and sequencing data are summarized in Table 1. The average number of reads mapped to the genome averaged 30 million (M) reads (22 M–38 M), with exon reads at 84.5% (82.2–86.7%) from total mapped reads across all 12 samples (Table 1). The proportion of globin mRNA was significantly higher (*p* < 0.05) in the NEBgr with 6.3% (±2.3%), while the other three kits were below 1% (Fig. 3a). All four kits showed successful removal of most rRNA with < 1% from the total mapped reads (Fig. 3b). The total junction reads were significantly higher in the probe hybridization depletion method GLOBINClear and GZr (37–40% from total mapped reads, *p* < 0.01) than enzymatic methods NEBgr and RZrs (25–36%, Fig. 3c). In addition, the gene body coverage plot showed that the probe hybridization method covered the entire gene body uniformly. In contrast, the enzymatic removal methods revealed skewed expression to the 3′ region of genes, indicating that RNA degradation likely occurred during the depletion step (Fig. 4).

**Table 1:**
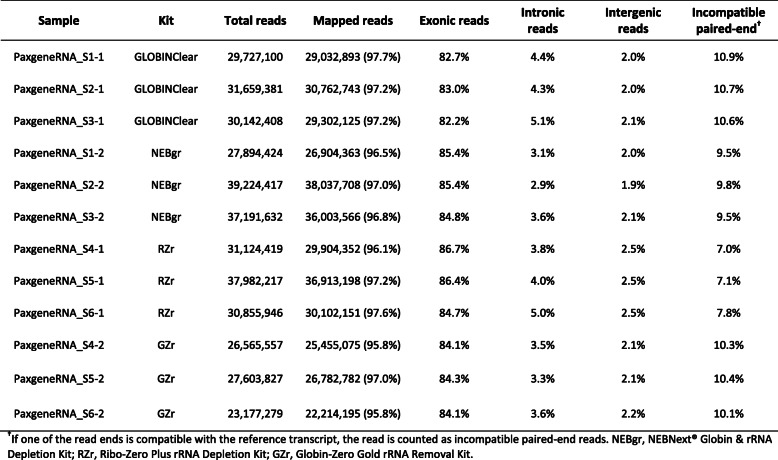
mRNA Sequencing data summary

**Fig. 5:**
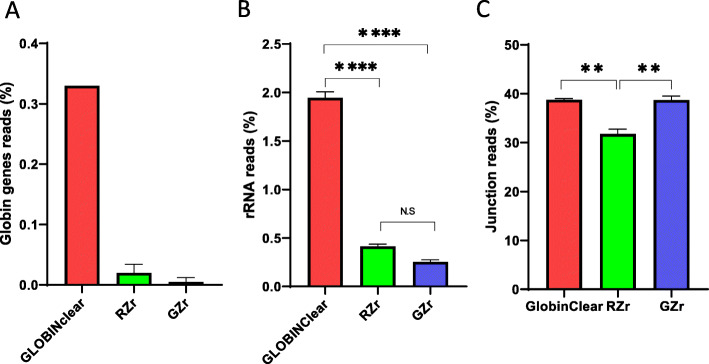
Comparison of globin gene, rRNA depletion, and junction reads among three kits. **a** Percentage of globin gene contamination in the total mapped reads, **b** Percentage of rRNA contamination in the total mapped reads, **c** Percentage of junction reads in the total mapped reads. Data are means of triplicate samples from each kit ± SD. **; *p* < 0.01, ****;*p* < 0.0001. RZr, Ribo-Zero Plus rRNA Depletion Kit; GZr, Globin-Zero Gold rRNA Removal Kit

Next, NEBgr was excluded from the second analysis because of the significant quantity of transcripts from globin genes remaining in the total reads. To permit direct comparison analysis among the kits, we made one RNA pool from six samples and performed depletion procedures with three kits. These samples were sequenced with average 56 M - 72 M reads mapping to the genome, exon reads were similar to those in the first dataset (81.8–86.3%, Table 2), and globin mRNA contamination rates were below 0.5% (Fig. 5a). The rRNA reads were significantly higher in the GLOBINClear (*p < 0.0001*) but still below 2% from the total mapped reads (Fig. 5b). As observed in the first data set, the probe hybridization method yielded more junction reads (38–39%) than enzymatic removal methods (31–32%, Fig. 5c).

**Table 2:**
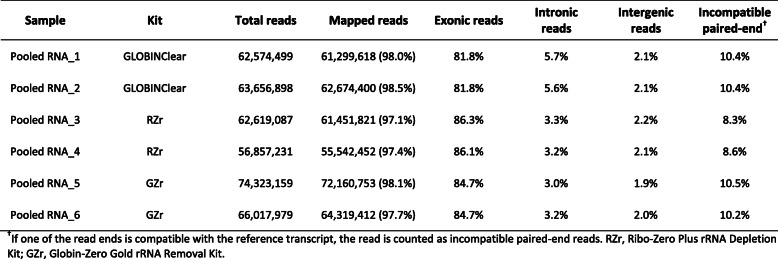
mRNA Sequencing data summary for the second set

**Fig. 6:**
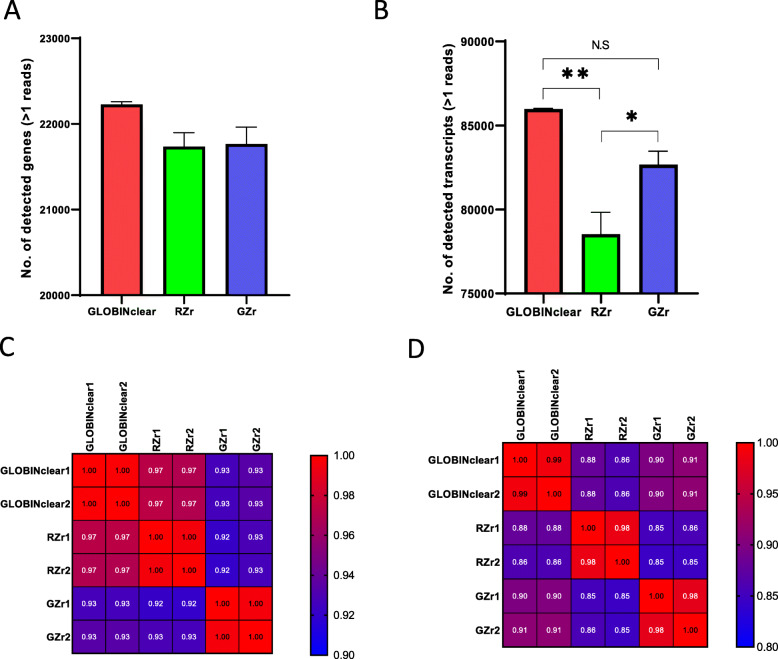
Comparison of detected genes and transcript and correlation among the tested kits. **a** The total number of detected genes, **b** The total number of detected transcripts, **c** Correlation values between samples using the total number of detected genes, **d** Correlation values between samples using the total number of detected transcripts. Data are means of triplicate samples from each kit ± SD. *; *p* < 0.05, **; *p* < 0.01. Pearson r values were used in each comparison. RZr, Ribo-Zero Plus rRNA Depletion Kit; GZr, Globin-Zero Gold rRNA Removal Kit; N. S, not significant

For direct comparison, the data were normalized with FPKM and transformed as log_2_ values to determine the sensitivity of each kit. At the gene level, the detected number of genes was not significantly different among the kits; GLOBINClear, 22,228 genes; RZr, 21,736 genes; GZr, 21,766 genes (Fig. 6a). However, at the transcript level, significantly more transcripts were detected in the GLOBINClear (85,979), with 78,526 transcripts observed in the RZr, and 82,669 transcripts in the GZr (Fig. 6b). In terms of data correlation between the kits at the gene level, GLOBINClear and GZr were highly correlated with the RZr, r > 0.97 and r > 0.93, respectively (Fig. 6c). Also, at the transcript level, a relatively high correlation (r > 0.90) was observed between GLOBINClear and GZr. In contrast, the RZr showed a moderate correlation to both GLOBINClear (r > 0.86) and GZr (r > 0.85, Fig. 6d).

## Discussion

Stranded mRNA-Seq was used to assess four globin gene depletion kits to allow a sensitive assessment of the detection of transcripts. Globin gene depletion from whole-blood derived RNA does reduce both the amount and quality of RNA [8] but is an essential procedure for global RNA-Seq analysis. In this study, we directly compared the performances of both probe hybridization and RNAse-H enzymatic depletion methods using four commercially available kits using mRNA Seq. Overall, the probe hybridization method showed a better performance with an increased total number of genes and transcripts detected without 3′ region bias seen with the enzymatic depletion methods.

Depletion approaches reduce RNA and also impart some degree of degradation, thus starting with higher purity and quantities of RNA ensures performance in downstream assays [8]. Adding a second DNaseI treatment step after RNA extraction from PAXgene Blood RNA Tubes enabled the generation of improved quality sequencing data, and the efficiency of depletion revealed removal of > 99% of globin genes in three of the four kits. While residual rRNA contamination was found in all tested samples ranging from 0.2–2% level of the total mapped reads, high-quality sequencing data mapping to the reference genome at > 96% of the total reads was generated, significantly better than previously reported (14–86%) [10, 12].

Among the Globin gene and rRNA removal kits, the probe hybridization method, GZr showed the lowest recovery yields likely related to the multiple cleanup steps required to remove the rRNA and globin genes. The RNase H-based RNA depletion, RZr, method was faster with higher recovery yields, and more streamlined processing than the probe hybridization method, with all enzymatic reactions carried out in a single tube. However, the combined RNase H and DNAseI enzyme activity did affect RNA quality and subsequently generated 3′ biased sequencing data, particularly in the longer transcripts. Overall, we observed that the RNase H-based RNA depletion method generated significantly fewer junction reads and a reduced number of total transcripts than the probe hybridization method. Therefore, due to the partial degradation of mRNA during the depletion step, RNase H-based RNA depletion may be a more appropriate method for the total RNA sequencing, which does not require poly-A+ selection.

Between the probe hybridization depletion method kits, GZr showed a reduced correlation than RZr when compared to GLOBINClear at the gene level. We assume that the total input of the depleted RNA for mRNA-seq library construction affects detecting the expression level of the lower copy of genes and transcripts between two kits; this may be the main cause of reduced correlation at the gene level between two kits as GZr tends to lose RNA during cleanup of the hybridized streptavidin beads. Also, GZr depletes both globin genes and rRNAs, including mitochondrial rRNA, therefore retains fewer amounts of depleted RNAs than GlobinClear that only depletes globin genes. Subsequently, poly-A selection is required at the beginning of the mRNA-Seq library construction procedure, which is a double negative selection of rRNA in the GZr group. However, as a result, GZr showed the best performance of the depletion of both rRNA and Globin genes from the total mapped reads. The GLOBINClear has a lower price and yielded more detected genes and transcripts than other kits. Thus, the probe hybridization depletion is an appropriate method for the mRNA sequencing that is both reliable for quantification and accurate for mature coding transcripts.

## Conclusions

In this study, we showed 1 μg of high-quality RNA from whole blood collected in PAXgene Blood RNA tubes may be routinely used for transcriptional profiling analysis studies. In addition, we have demonstrated that the probe hybridization depletion method is more suited to mRNA sequencing analysis with minimal effect on RNA quality during depletion procedures from whole blood-derived RNA. Therefore, our results should help biobanking efforts that allow us to do more affordable mRNA sequencing with high resolution of transcriptome profile study of whole blood.

## Methods

### Total RNA extraction from whole blood

Peripheral whole blood samples from six volunteers were collected in PAXgene Blood RNA tubes (PreAnalytiX GmbH, BD Biosciences, Mississauga, ON, Canada) following institutionally approved IRBs. Total RNA was extracted from four aliquots using a PAXgene Blood RNA Kit with DNaseI treatment (Qiagen, Chatsworth, CA, USA) according to the manufacturer’s protocol. The extracted RNA was cleaned with DNAseI using Zymo RNA Clean and Concentrator Kit (Zymo Research, CA, USA), and yield and quality of the purified RNAs were evaluated using a Qubit (Thermo Fisher Scientific, MA, USA) and Agilent 2100 Bioanalyzer (Agilent Technologies, Santa Clara, CA, USA), respectively. Those RNAs with RIN values > 7.5 proceeded to the globin mRNA and rRNA removal step from total RNA. Figure 1 outlines the major steps of this study.

### Globin gene depletion

A total of 1 μg of each RNA sample was further processed with a GLOBINClear Kit (Thermo Fisher Scientific, MA, USA) to remove globin mRNA. Briefly, to remove globin mRNA using this kit, a total of 1 μg of RNA was mixed with a biotinylated capture oligo mix and incubated for 15 min to hybridize with the globin mRNA. Washed streptavidin magnetic beads were added and incubated for 30 min, then the beads were pulled to the bottom of the plate on a magnet plate, and globin-depleted mRNA was transferred to a new tube. Another 1 μg of total RNA was used for both globin mRNA and rRNA (cytoplasmic and mitochondrial) depletion using NEBNext® Globin & rRNA Depletion Kit (NEBgr) (NEB, MA, USA), Ribo-Zero Plus rRNA Depletion Kit (RZr) (Illumina, CA, USA), or Globin-Zero Gold rRNA Removal Kit (GZr) (Illumina, CA, USA), according to the manufacturer’s protocols with some modifications. For GZr, total RNA was mixed with biotinylated oligonucleotides mixture, and the streptavidin magnetic beads were added and incubated for binding to the hybridized mixture. The streptavidin magnetic beads were pulled down to the bottom of the plate on a magnet plate, and the depleted RNA was transferred to the new plate for the next RNA cleanup step. The NEBgr and RZr employ enzymatic depletion to remove globin mRNA and rRNA, and both kits have a similar procedure. Briefly, total RNA was hybridized with single-strand DNA probes, and RNase H digestion was performed. To remove the excessive single-strand DNA probes, DNaseI treatment was carried out after RNAase H digestion. All depleted RNAs were mixed with 1.8 times amounts of Ampure RNA Clean XP beads (Beckman Coulter, IN, USA) for the enrichment and eluted with 50 μl of water. Then, 1 μl of each RNA was assessed with an Agilent 2100 Bioanalyzer using an RNA 6000 Pico Chip to verify the depletion of globin mRNA and rRNA. The RNA was stored at − 80 °C before being analyzed.

### mRNA-Seq library construction and data analysis

The TrueSeq Stranded mRNA-Seq Kit (Illumina, CA, USA) with poly-A+ selection was used to generate the mRNA-Seq library from depleted RNA samples using four different kits, according to the manufacturer’s protocol. Constructed libraries were quantified by BioAnalizer 2100 system using the D1000 kit (Agilent) and Qubit dsDNA BR Assay kits (Thermo Fisher Scientific). All libraries were pooled (12 samples, first set; 6 samples, second set) and sequenced 101 bp paired-end reads on Illumina HiSeq 4000. Approximately 30 to 60 million (M) pairs of total reads were generated from each library. FASTQ files were uploaded into Partek Flow software (Partek Inc., MO, USA), and primary QC was performed. The STAR (2.6.1d) aligner was used to align reads to the human reference genome (hg38). After alignment, the final BAM files were quantified using Partek E/M algorithm [13] by Ensembl annotations (Ensembl Transcripts release 92) and then normalized to FPKM (fragments per kilobase of transcript per million mapped reads) values. Also, the BAM files were used for globin gene and rRNA quantification, calculated using the percentage of the total mapped reads using Partek E/M algorithm. For sample correlation analysis, normalized expression values (log_2_-transformed FPKM + 1) were used. Sequence mapping to the genome and the transcriptome was visualized in Integrative Genomics Viewer (IGV, Broad Institute). The one-way ANOVA test was used for all statistical analyses (GraphPad Prism 8.0, GraphPad Software), and *p*-value < 0.05 was considered as statistically significant. Pearson *r* value was used for correlation analysis of mRNA-Seq data from each kit.

## Data Availability

The sequencing datasets analyzed during the current study were deposited in the GEO repository (GSE150587, http://www.ncbi.nlm.nih.gov/geo/query/acc.cgi?acc=GSE150587).

## Abbreviations

mRNA-Seq: mRNA sequencing
GLOBINClear: GLOBINClear Kit
NEBgr: NEBNext® Globin & rRNA Depletion Kit
RZr: Ribo-Zero Plus rRNA Depletion Kit
GZr: Globin-Zero Gold rRNA Removal Kit
rRNA: Ribosomal RNA
FPKM: Fragments per kilobase of transcript per million mapped reads
